# Believing in or Denying Climate Change for Questionable Reasons: Generic Conspiracist Beliefs, Personality, and Climate Change Perceptions of Romanian University Students

**DOI:** 10.3390/ijerph192417038

**Published:** 2022-12-19

**Authors:** Ștefan Boncu, Oara Prundeanu, Andrei Corneliu Holman, Simona Andreea Popușoi

**Affiliations:** Psychology Department, Faculty of Psychology and Educational Science, Alexandru Ioan Cuza University of Iași, 700506 Iași, Romania

**Keywords:** climate change beliefs, conspiracy beliefs, personality, Big Five

## Abstract

People’s perceptions of climate change represent a growing concern, especially when these perceptions entail the denial of climate change. Past studies have highlighted the detrimental role of conspiracist beliefs concerning climate change regarding people’s perceptions on this matter. However, the effects of generic conspiracy beliefs and the different types of beliefs determining skepticism about climate change, as well as that of an individual’s personality, are still an open area of inquiry. Our cross-sectional study (N = 842) explored the relationships between the degree to which people hold different generic conspiracy beliefs, their personality characteristics (as defined within the Big Five taxonomy), and climate change beliefs (i.e., in its occurrence and anthropogenic causation). Results indicated common predictors of these dimensions of climate change beliefs, specifically three of the five types of generic conspiracy beliefs, extraversion, agreeability, and intellect/imagination as personality factors. While conspiracy beliefs related to personal well-being emerged as related to climate change skepticism, those in government malfeasance and information control were found to be associated with more acceptance of climate change and its anthropogenic causation. These findings reveal a mixed pattern of relationships between different conspiracist beliefs and climate change perceptions and suggest the complex psychological and ideological underpinnings of the attitudes towards climate change.

## 1. Introduction

Human-caused climate change has widespread negative effects associated with nature and humans, beyond the natural variability of climate. The Intergovernmental Panel on Climate Change (2022) highlighted the fact that people and systems are disproportionately affected by climate change damage, and the level of risk will depend on competing short-term trends in terms of exposure, development, and adaptation. A recent global survey of 10,000 young people from ten countries indicated that 59% were extremely worried about climate change, and 75% considered their future to be frightening [1]. 

Results from a cross-sectional survey conducted in 2007 and 2008 on representative samples in 119 countries indicated that the strongest predictor of climate change risk perceptions in Latin America and Europe is the understanding of the anthropogenic cause of climate change [2]. However, not all people believe that climate change is real, and conspiracy theories are one important factor. Even though climate change can adversely affect the health and well-being of individuals [3], exposure to conspiracy theories reduces science acceptance [4], and conspiracy thinking predisposes to climate change denial [5,6] and to less concern about issues that are specific to climate change [7]. Beliefs in climate change conspiracy theories are also related to reticence towards the solutions proposed to reduce the negative impact of humans on the environment [8] and to lower the support for pro-climate policies [9]. Moreover, individual differences on specific personality traits can have important roles in predicting whether an individual will develop positive or negative climate change attitudes or is inclined to be interested in climate change issues [10,11]. This study examines the links between generic conspiracy beliefs, personality traits, and climate change beliefs.

### 1.1. Dimensions of Climate Change Beliefs and Skepticism

As we face multiple problems related to climate change, it is necessary to analyze more deeply the beliefs that people have about this important phenomenon. These beliefs can range from complete awareness of climate change, its anthropogenic cause, and its detrimental impact to skepticism about or denial of these issues. In [12], a framework was proposed that distinguishes between three types of skepticism about global warming, which was later used in investigations on the public beliefs of the broader phenomenon of climate change. Trend skepticism is oriented towards the actual occurrence of climate change. Attribution skepticism entails the belief that any change in the world’s climate is not caused by human activity. In this respect, some people may consider that certain changes have anthropogenic causation, being generated by human activities that produce negative effects on the environment, while others consider that these changes are due to the natural cycle of the environment or the normal course of events [13]. Finally, impact skepticism refers to the belief that climate change will not have significant detrimental effects. This framework has been used in various studies on the ways people represent climate change. For instance, the “Climate Change in the American Mind” US surveys (e.g., 13) have examined whether people believe that global warming is happening (the trend dimension), is mostly human-caused or not (the attribution dimension), and whether it generates significant risks and harm to them, others and the environment (the impact dimension). The authors of [14] performed an investigation of skepticism about climate change nationally representative quota sample of the British population and examined the relationships between the three dimensions of climate change skepticism of Rahmstorf’s framework to participants’ socio-demographic, attitudinal and ideological characteristics. Their results also suggested strong links between the different types of climate skepticism, as skepticism on one dimension of this framework is associated to skepticism about the other aspects of climate change.

Our study used a measure of climate change beliefs developed by [15] and tested across other investigations in different cultural contexts [16,17]. The scale addresses two of the dimensions in the framework proposed by [13], i.e., people’s beliefs about climate change occurrence and the attributions individuals make to explain climate change.

### 1.2. Climate Change Skepticism among the Romanian Population

People’s awareness of and beliefs about climate change vary considerably across countries worldwide [2]. The latest Special Eurobarometer report [18] highlights the large proportion of climate change skepticism among the Romanian population in comparison to the other European Union (EU) countries. Specifically, while 18% of the EU residents in average consider climate change to be the single most serious problem facing the world, only 7% of the Romanian respondents endorse this idea, while 66% others agree that climate change is a very serious problem, considerably below the EU average of 78%. Furthermore, Romanian residents are the least likely (31%) in any EU country to have taken action to fight climate change in the last six months, this percentage being less than half than the EU average (64%). This low involvement in tackling climate change corresponds to the tendency of Romanian residents to perceive this environmental issue as outside their responsibility, as only 26% consider themselves as personally responsible for actions that would combat climate change, far below the EU average of 41%.

This pattern of low personal involvement and underestimation of the seriousness of the issue of climate change, albeit with a lower magnitude, has also emerged in studies on other Eastern European countries [19,20], and it has received multifaceted explanations. Firstly, the rapid structural changes of the post-communist societies may have pushed environmental protection at the end of the list of priorities for the majority of citizens of these countries, economic adaptation and survival being far more important in their personal agenda [20]. In comparison to the severe economic challenges that people in these societies have had to cope with, climate change has appeared as an abstract and remote threat [21]. Moreover, a cultural lag in environmental attitudes in general in comparison to the Western cultures has also been invoked as a complementary explanation [20]. Past research on Romanian samples have also suggested that economic security is the dominant priority of most of the population, a tendency which is also associated to that of focusing on local threats while mostly ignoring global ones [22,23]. Both these country-level specificities render climate change as an issue of low concern for the Romanian public in comparison to many EU countries.

Furthermore, the Eurobarometer data and other investigations indicate that in most European countries young respondents have a stronger tendency to consider climate change as a highly severe problem [24,25]. Yet, this association between age and climate change concerns does not appear in the case of the Romanian population, as the 2019 Special Eurobarometer data reveals [23]. Moreover, a recent investigation [26] of the perceived importance of environmental issues found the opposite relationship in a Romanian sample, with a lower percentage of the young participants considering environmental issues to be very important in comparison to the other age groups. It is plausible that the general tendency of the young populations worldwide to be more sensitive about environmental and climate change issues to be, in the case of the Romanian youth, attenuated by opposing drivers of their beliefs on these matters, which make them more skeptical about the scientific evidence on climate change compared to their similar age counterparts in the rest of the EU. Therefore, considering the specificities of the Romanian residents’ perceptions of environmental issues in general and of climate change in particular that have been indicated by past findings, our study aimed to further explore the associations of climate change skepticism in a sample of young people from this population.

### 1.3. Conspiracy Beliefs and Climate Change Beliefs

The uncertainty surrounds public understanding of climate change provides fertile ground for adherence to certain conspiratorial beliefs. Thus, understanding the psychological factors of climate change skepticism can help us overcome certain barriers that may arise in the way of positive actions towards the environment. Ref. [27] defined conspiracy as “a secret arrangement between a small group of actors to usurp political or economic power, violate established rights, hide vital secrets, or illicitly cause widespread harm”. Explaining events by invoking certain groups that act secretly only for their own benefit and against the good of others can be understood as a conspiracy theory [27]. Thus, conspiracy beliefs have the root in specific conspiracy theories [28]. Therefore, conspiracy beliefs can be defined as “attempts to explain the ultimate causes of significant social and political events and circumstances with claims of secret plots by two or more powerful actors” [29].

People who reject the scientific consensus that humans are causing climate change are described as climate deniers [30]. The denial of the scientific consensus and the doubts of individuals related to climate change often led to inaction, thus, to negative environmental and social effects [31]. Ref. [9] found in their meta-analysis performed on 22 independent samples that climate change conspiracy beliefs are negatively linked with trust and acceptance of climate science, and hinder climate change mitigation initiatives. The conspiracy theories related to the topic of climate change include, among others, ideas such as that it is merely a hoax, based on systematic scientific fraud, that climate change is a component of a sinister plot implemented globally by elites, governments or secret organizations aiming to create a New World Order, or that the changes of climate are in fact produced by powerful agents using advanced geoengineering [9]. Adherence to such beliefs can undermine individual’s trust in the scientific evidence related to climate change and in its accounts based on this evidence. Individuals’ exposure to conspiracy theories may also influence their intention to reduce their carbon footprint, a relationship that can be explained by climate powerlessness or uncertainty [32]. Hence, past studies suggest that conspiracy beliefs relate to climate change skepticism and that the more individuals engage in conspiracy thinking, the more likely they are to reject the scientific consensus on climate change [33]. Therefore, we expect that conspiracy beliefs positively affect climate change skepticism (i.e., occurrence and anthropogenic causation).

### 1.4. Personality and Climate Change Beliefs

Past research has already found that demographic characteristics, socio-economic status, and psychological traits are important in explaining pro-environmental actions [34,35]. Some models that analyze environmental behaviors highlight the fact that personality is at the root of individual differences, mostly because it represents a core part of what motivates our beliefs, values, and attitudes [36,37]. Personality traits can have a positive role in enhancing the impact of attitudinal factors on individual pro-environmental behaviors in response to climate change [38] and are related to pro-ecological environmental intentions [39]. A recent meta-analysis [40] concluded that openness, honesty-humility, agreeableness, conscientiousness, and extraversion were positively associated with pro-environmental attitudes and behaviors. Furthermore, agreeableness, conscientiousness and openness to experience were linked to environmental engagement [41] and environmental concern [42]. Also, some personality traits could be related to climate change skepticism. For example, individuals with low tolerance of ambiguity tend to deny anthropogenic climate change, an effect that may stem from their preference for more familiar and simpler (thus less complex and ambiguous) explanations, such as that natural fluctuations of weather are responsible for what science now defines as “climate change” [43].

From the Big Five perspective [44], Openness represents the personality trait that demonstrated a positive relational pattern with various pro-environmental behaviors [40,45,46] and environmental concern [42]. Some authors label Openness/Intellect (i.e., the individual’s tendency to be curious, imaginative, perceptive, artistic, clever, and intelligent) when focusing on Big Five aspects because Openness and Intellect are two highly correlated facets of a broader domain that is usually labelled as Openness to experience [47]. Agreeableness (i.e., the individual’s tendency to show compassion, generosity, and to cooperate with others or offer help when they need it) represents another personality trait strongly associated with environmental engagement [41], environmentalism [48] and climate change acceptance [45]. Concerning the latter, both Openness and Agreeableness predispose to stronger perception of relatedness with nature, i.e., the tendency to define oneself as integrated with the natural world [40,45,49]. In turn, this ecological self-concept may induce a greater sensitivity towards the endangerment of nature, and thus more attention and receptivity towards the scientific messages on the topic of climate change. Furthermore, Openness also fosters flexible and abstract thinking, important for representing the phenomenon and the long-term consequences of climate change [10]. Thus, we expected that both intellect and agreeableness will positively affect climate change acceptance.

Regarding extraversion, conscientiousness, and neuroticism, previous studies have obtained mixed results. Ref. [48] found that none of these traits was significant predictors for environmentalism or environmental concern, while ref. [50] reported that tourists with higher levels of extraversion, conscientiousness, and neuroticism manifest more pro-environmental behaviors. Extraverted individuals have a friendly and expansive attitude, are ready to take risks and often take the initiative in dealing with others. People who score high on conscientiousness are ambitious, organized, and meticulous, confident in their personal ability to handle unexpected problems. Individuals with a high level of neuroticism have a low emotional balance, being often worried, anxious, and tense, experiencing negative affects such as irritability, fear, or anger. Ref. [11] analyzed the influence of personality traits on climate change belief certainty and risk perception and found that extraversion, conscientiousness, and neuroticism are not significantly associated with attitudes towards climate change. However, other results indicated that the strongest predictors of motivated reasoning in anthropogenic climate change were higher emotional stability and dogmatism [51]. Despite these mixed results, there is consistent evidence that several Big Five personality traits can be factors of climate change beliefs [52]. Thus, we expected that extraversion, conscientiousness, and emotional stability positively affect climate change beliefs.

The aim of this study was to investigate the relationships between conspiratorial beliefs, Big Five personality factors and climate change beliefs. While most past research revealed that conspiracist thinking in general predisposes to climate change skepticism, among other effects on environmental attitudes and behaviors, our study aims to use a more fine-grained grid of analysis of conspiratorial beliefs in relation to perceptions of climate change occurrence and human causation. Specifically, we examined the effects of different types of conspiratorial beliefs on climate change perceptions in order to highlight the most important determinants of climate change skepticism among these distinct facets of conspiracist thinking.

## 2. Materials and Methods

### 2.1. Participants and Procedure

A convenience sample of 842 (51% women) participants took part in this study. Most participants were undergraduate students (62% bachelor’s degree studies) from a university in the north-eastern part of Romania. Their age ranged from 18 to 25 (M = 20.39; SD = 1.79) and most of them were from an urban area (65.8%). Participants were recruited by students in exchange for course credits. Students were instructed to identify among personal acquaintances at least four people with the minimum age of 18 years and to send them an online questionnaire form. All participants received an informed consent form about the scope of the study, the confidentiality of their responses, their rights to withdraw from the study and the average time for completing the questionnaires (15–20 min).

### 2.2. Measures

#### 2.2.1. Personality

To measure Big Five Factor Markers, we used the Romanian version of The International Personality Item Pool (IPIP), adapted by [53]. We used five scales, each containing ten items, that measure extraversion (e.g., “talk to a lot of different people at parties.”), agreeableness (e.g., “take time out for others”), conscientiousness (e.g., “follow a schedule”), emotional stability (e.g., “I am relaxed most of the time”) and intellect or imagination (e.g., “spend time reflecting on things”). Participants were asked to rate how well each statement described them using a 5-point Likert scale, where 1—strongly disagree and 5—strongly agree. The internal consistency of these scales was high (see Table 1).

#### 2.2.2. Conspiracist Beliefs

We used The Generic Conspiracist Beliefs Scale (GCB) [54], a 15-item instrument to measure conspiracy ideation. This scale contains five types of conspiracy beliefs with three corresponding items each: government malfeasance (e.g., “the government permits or perpetrates acts of terrorism on its own soil, disguising its involvement”); extraterrestrial cover-up (e.g., “evidence of alien contact is being concealed from the public”); malevolent global conspiracies (e.g., “a small, secret group of people is responsible for making all major world decisions, such as going to war”); personal wellbeing (e.g., “technology with mind-control capacities is used on people without their knowledge”); and control information conspiracies (e.g., “new and advanced technology which would harm current industry is being suppressed”). Participants rated items on 5-point Likert scale, where 1—definitely not true and 5—definitely true. Higher scores on this instrument represent higher levels of generic conspiracist ideation and the internal consistency of these scales was high (see Table 1).

#### 2.2.3. Climate Change Beliefs

The Occurrence and Anthropogenic Causation Scale (OC-AN) [55] represents a short scale composed of 14 items that captures the most frequently raised and already established issues in the literature on the skepticism about climate change: belief that climate change exists and is currently happening (i.e., occurrence) and belief that climate change is mainly caused by human activities (anthropogenic causation). Participants rated items on a 7-point Likert scale, where 1—completely disagree and 7—completely agree (e.g., “on average around the earth, I believe the following are happening … the temperature of the ocean is increasing”—for occurrence dimension; “I believe the following contribute to changes in climate around the earth … clear cutting of forests”—for anthropogenic causation).

#### 2.2.4. Demographics

Participants were asked to report their age, gender, study level and residence.

## 3. Results

We tested all the variables for normality, considering the range values for Skewness and Kurtosis, namely ±2 [56]. Summary statistics for all the variables are presented in Table 1.

Zero-order correlations between personality traits and climate change beliefs indicated that agreeableness and intellect were positively and significantly correlated with belief in the occurrence of climate change, but also with belief in the anthropogenic causes of climate change. Emotional stability correlated negatively and significantly only with beliefs in anthropogenic causation of climate change. There were no statistically significant correlations between extraversion, conscientiousness, and the dimensions of climate change beliefs. The results of the analysis of the correlation between the dimensions of general conspiracy beliefs and climate change beliefs showed that there are negative, significant correlations between the dimensions of extraterrestrial cover-up, global conspiracies, personal well-being, and belief in the anthropogenic causes of climate change. Only the dimension of government malfeasance correlated positively and significantly with occurrence of climate change beliefs. In terms of socio-demographic variables, age positively and significantly correlated with government malfeasance, global conspiracy, personal well-being, and informational control of general conspiracy beliefs. Women scored higher on the belief in the anthropogenic causes of climate change, and men scored higher on extraterrestrial coverage of general conspiracy beliefs (see Table 2). All these correlations had a low magnitude (i.e., below 0.3), except the association between agreeableness and anthropogenic causation beliefs, which had a moderate magnitude.

Next, we used hierarchical multiple regression analyses to examine the specific effects of personality traits and general conspiracy beliefs on the dimensions of climate change (i.e., occurrence beliefs and anthropogenic causation beliefs), while controlling age and gender (see Table 3). Before performing this analysis, we checked whether the conditions required by the hierarchical multiple regression are met. Although some independent variables were highly correlated (see Table 2), the collinearity statistics (i.e., Tolerance and VIF) were all within acceptable limits, thus the assumption of multicollinearity was met [57]. Residual and scatter plots indicated the relevant assumptions were reasonably satisfied. 

The first regression analysis was performed on the belief in the occurrence of climate change as criterion and included personality traits, gender, and age in the first block, and the five types of general conspiracy beliefs were added in the second block. The results indicated that the model that contains all the predictors was statistically significant (*F* (12, 829) = 10.65, *p* < 0.001). Gender was a negative predictor (*β* = −0.08, *p* < 0.05), while age (*β* = −0.01, *p* = 0.67) was not a significant predictor. In terms of personality traits, agreeableness (*β* = 0.22, *p* < 0.001) and imagination/intellect (*β* = 0.15, *p* < 0.001) positively predicted the belief in the occurrence of climate change, whereas extraversion (*β* = −0.09, *p* < 0.05) negatively predicted occurrence beliefs. Regarding the five types of conspiracy beliefs, the results indicated that government malfeasance (*β* = 0.24, *p* < 0.001), and information control (*β* = 0.14, *p* < 0.01) were positive predictors of belief in the occurrence of climate change, while personal well-being conspiracy belief was a negative predictor (*β* = −0.24, *p* < 0.001). Overall, 12% of the variation in occurrence beliefs was explained by the predictors included in the second model.

The second regression analysis was performed on anthropogenic causation beliefs of climate change as criterion, with the same predictors as in the first. The model that contains all the predictors was statistically significant (*F* (12, 829) = 13.22, *p* < 0.001). Gender (*β* = 0.00, *p* = 0.99) and age (*β* = 0.01, *p* = 0.66) did not significantly predict anthropogenic causation beliefs. Regarding personality traits, the pattern of results was similar to that found in the first regression analysis. Agreeableness (*β* = 0.29, *p* < 0.001) and imagination/intellect (*β* = 0.13, *p* < 0.001) positively predicted the belief in the anthropogenic causation of climate change, whereas extraversion (*β* = −0.12, *p* < 0.001) negatively predicted anthropogenic causation beliefs. Government malfeasance (*β* = 0.10, *p* < 0.05), and information control (*β* = 0.16, *p* <.01) were positive predictors of anthropogenic causation beliefs of climate change, while personal well-being conspiracy belief was a negative predictor (*β* = −0.23, *p* < 0.001). The predictors in the second model explained 14% of the variation in anthropogenic causation beliefs.

## 4. Discussion

The present study explored the relationships between the degree to which people hold different types of generic conspiratorial beliefs, their personality characteristics examined through the Big Five taxonomy, and perceptions of climate change on two dimensions (the actual occurrence of climate change and its anthropogenic causality). The result of the regression analyzes revealed common predictors of these dimensions of climate change beliefs, specifically three general conspiracy beliefs (i.e., government malfeasance, personal well-being, and information control), as well as three personality factors (i.e., extraversion, agreeableness, and intellect/imagination). These findings partially confirmed the proposed research hypotheses and suggest specific patterns of relationships between certain conspiracy beliefs and personality, on the one hand, and perceptions of climate change, on the other.

While past research examined the general relationship between adherence to conspiracy theories and climate change skepticism, the results of our more fine-grained analysis indicate that some of the specific types of such beliefs are more strongly associated to climate change perceptions than others. Furthermore, we found that there are certain conspiracy beliefs (i.e., those in government malfeasance and information control) who make people more prone to accept climate change and its anthropogenic causation as true, while others (i.e., personal well-being) have the opposite effect of inducing disbelief in these ideas. The latter result is similar to the associations between general conspiracist thinking and climate change skepticism highlighted by past research [5,6]. 

Firstly, our results indicated that people who believe that governments are responsible for certain covert or criminal actions directed against citizens or that that there is increased control of public information and manipulation by various organizations report stronger beliefs regarding the occurrence of climate change and its anthropogenic causality. As past research indicated, individuals who are prone to conspiracist thinking tend to reject official accounts and distrust authorities [58,59]. In the realm of climate change issues, people who hold these two types of generic conspiracy beliefs manifest these tendencies not through skepticism about the occurrence of this phenomenon, but through suspicions and alternative interpretations focused on the “real” interests of powerful actors involved in the change of the world’s climate. Therefore, people who hold such conspiracist beliefs and consider that climate change exists and is determined by human actions may have a specific way of understanding this human causation in comparison to most of the general public, by focusing on external and powerful agents whom they blame for climate change. These individuals may feel that there are certain technologies that could be implemented to reduce climate change, but they are kept hidden or discouraged because their large-scale use would be detrimental to the economic or political interests of certain influential organizations. Consequently, these ideas can cause people to be inert about pro-environmental actions because they believe that other social actors are responsible for the problems we face, and thus they also have the moral obligation to solve them, in line with past findings on the way some people make external attributions for environmental issues [60]. The principle “they did it with their own hands, now they just have to fix it” could be the way people with these conspiratorial beliefs relate to climate change issues. 

Secondly, the negative association between conspiratorial beliefs related to personal well-being and climate change perceptions indicates that people who tend to think that certain secret organizations use various technologies on people without their knowledge and with deleterious health effects are more prone to deny climate change and its anthropogenic causes. This may suggest that the tendency to accept certain conspiracy theories that relate to individual health and well-being may predispose individuals to deny scientific consensus. This result is in line with past evidence indicating that in general, conspiratorial thinking may contribute to the rejection of the scientific consensus on climate change [33], and that exposure to conspiracy theories reduces individuals’ engagement in pro-environmental behaviors and their acceptance of scientific evidence [4]. The positive relationship between this specific type of generic conspiracy beliefs and climate change skepticism suggests that in people who hold these beliefs their tendency to reject official accounts and distrust authorities generates an opposite pattern of representing issues related to climate change. Specifically, suspecting that technologies are used for secret and malevolent purposes at the expense of people’s health renders plausible the idea that climate change is a “hoax” perpetrated in order to deter public attention from these technologically—mediated abuses and maybe to create a scapegoat that would cover the real source of their deleterious effects on public health. The feeling of powerlessness highlighted by past research [32] as an effect of conspiracist beliefs maybe also important in motivating skepticism about climate change in individuals holding these beliefs, which place them as passive recipients or victims of external interventions from powerful agents who have the ability to manipulate the public agenda, inclusively by inventing fake threats such as climate change.

Considering the specific population of the study, this pattern of relationships indicated by our results suggest that young Romanian residents’ personal position towards the topic of climate change may be influenced, at least in part, by their adherence to certain generic conspiracist theories centered on hidden but impactful actions of powerful agents aiming to attain their secret goals [27]. Past findings have highlighted that the Romanian population tends to underestimate the seriousness of the issue of climate change and has a low involvement in tackling it, in comparison to the EU average, and that the Romanian youth does not show the higher concern about climate change of most young populations in other countries [18,23,25,26]. Our findings highlight conspiracist beliefs related to personal well-being as a significant factor that may affect young Romanians’ climate change beliefs, although the results of the correlational analysis also indicate other types of generic conspiracist theories which have negative association with these beliefs and thus fuel skepticism about climate change. Past research has found that climate change perceptions of Eastern European populations have weaker associations to personal values and political orientation in comparison to the other European countries [19,61]. This lower dependence of climate change beliefs on ideological factors may allow other drivers, such as generic conspiracist ideas, to exert a greater influence on these beliefs among the Eastern European residents. Future research could examine the relationships between the different generic conspiracist beliefs and climate change skepticism in young people of other countries, as well as develop cross-cultural comparisons aiming to identify the extent to which these beliefs are responsible for inducing skepticism about climate change. Regarding the relationships between personality and climate change perceptions, our results suggested that beliefs in both the occurrence of climate change and in its anthropogenic causality are stronger in people who favor cooperation and social harmony, and show compassion often (i.e., agreeableness) and in those who have a diverse range of interests, being intellectually curious (i.e., intellect/imagination). Similar results were obtained in previous studies that concluded that beliefs in climate change are stronger in individuals who hold altruistic values and who have high levels of agreeableness and openness to experience [11,38], while climate change skeptics have lower levels of agreeableness [62]. On the other hand, our findings indicate that people with high extraversion tend to believe that climate change is not real and that it is caused only to a small extent by human actions. While other studies did not find significant associations between extraversion and climate change beliefs [11], the relationship that emerged in our investigation may be due to the fact that extraverted people in our sample also hold beliefs in plots that threaten one’s personal wellbeing, as indicated by the results of the correlation analysis. As discussed above, this type of conspiratorial beliefs tends to predispose to climate change skepticism. Furthermore, conscientiousness and emotional stability traits did not predict climate change beliefs, a result that is consistent with other studies in the literature [63].

The results of this research contribute to the understanding of the psychological barriers that support the gap between scientific evidence and public perceptions about global environmental change by examining the relationships between personality traits, the most frequent generic conspiratorial beliefs and the acceptance of or skepticism about climate change. Generally, conspiratorial beliefs can block individuals’ willingness to engage in pro-environmental behaviors that can contribute to ameliorating aspects of climate change. Climate change skepticism and conspiracy ideation, along with science distrust, are negatively associated with pro-environmental attitudes and behaviors or support for pro-environmental initiatives [7]. At the same time, there is data indicating that not all climate change skeptics are conspiracy oriented or have no interest in the environment at all [63], in line with our results that highlighted mixed associations between climate change and conspiratorial beliefs. Future studies could investigate more closely the way in which people tend to explain the human contribution to climate change, as some individuals may believe in the existence of this phenomenon but consider themselves as having no responsibility for its occurrence or future mitigation. 

One of the limits of this study is related to the fact that it investigated a university student sample, thus its results cannot be generalized to the general population, as past research has indicated that climate change beliefs vary among Romanian people with different education levels [23]. However, it is important to analyze students’ beliefs about accepting climate change because they represent the generation that is likely to face the most significant problems related to climate change. Another important limit of our study is that it didn’t include a measure of skepticism about the impact of climate change, the third dimension in the framework proposed by [12]. However, the 2021 Special Eurobarometer data suggest that the Romanian population also has a high level of impact skepticism. For instance, the Romanian respondents were found to be the least likely in any EU country to agree with the statement that “the cost of damage due to climate change is much higher than the investment needed for a green transition”, indicating that the impact of climate change is more frequently underestimated among Romanian residents, at least in comparison to other EU populations. Further research should examine the relationships between generic conspiracy beliefs, personality and climate change beliefs in other cultural spaces, especially in those in which the young population has been found to have less concern about climate change. Other limits of our study are the reliance on self-report instruments, and the cross-sectional design of the study that does not provide information related to the way in which beliefs related to climate change can change over time.

## 5. Conclusions

The current study extends previous research by examining the effects of specific conspiracist beliefs and personality factors on climate change acceptance in a large sample. In regard to personality, we found that cooperative, compassionate and intellectually curious people are more prone to believe in both the occurrence of climate change and in its anthropogenic causality. Our findings also revealed a mixed pattern of relationships between conspiratorial and climate change beliefs, which indicates that certain conspiracist theories predispose to skepticism about climate change and about its anthropogenic causation, while others have the opposite effect. This highlights the complexity of the relationships between conspiracist thinking and climate change perceptions.

## Figures and Tables

**Table 1:** Descriptive statistics of the main study variables.

	Alpha	Mean Inter-Item Correlation	Min.	Max.	*M*	*SD*	Skewness (*SE*)	Kurtosis (*SE*)
*Personality*								
Extraversion	0.89	0.44	9.10	45.50	30.70	8.23	−0.21 (0.08)	−0.42 (0.16)
Agreeableness	0.86	0.40	10.10	45.50	36.65	6.46	−0.58 (0.08)	0.11 (0.16)
Conscientiousness	0.78	0.26	9.10	45.50	30.75	6.33	−0.04 (0.08)	−0.04 (0.16)
Emotional Stability	0.88	0.43	9.10	45.50	25.43	7.99	0.13 (0.08)	−0.45 (0.16)
Intellect/Imagination	0.81	0.31	14.10	45.50	33.79	5.78	−0.05 (0.08)	−0.32 (0.16)
*Generic Conspiracist Beliefs*								
Government malfeasance	0.77	0.53	2.33	11.67	6.36	2.61	0.20 (0.08)	−0.74 (0.16)
Extraterrestrial cover-up	0.81	0.60	2.33	11.67	4.86	2.555	0.79 (0.08)	−0.28 (0.16)
Malevolent global conspiracies	0.84	0.64	2.33	11.67	6.07	2.64	0.24 (0.08)	−0.77 (0.16)
Personal wellbeing	0.80	0.58	2.33	11.67	5.86	2.63	0.38 (0.08)	−0.66 (0.16)
Information control	0.72	0.46	2.33	11.67	6.71	2.44	−0.05 (0.08)	−0.58 (0.16)
*Climate change beliefs*								
Occurrence beliefs	0.91	0.58	7.13	49.88	40.85	7.98	−0.79 (0.08)	0.46 (0.16)
Anthropogenic causation	0.84	0.50	5.17	36.17	30.09	5.99	−1.24 (0.08)	1.50 (0.16)

**Table 2:** Inter-correlations between study variables.

Variables	1	2	3	4	5	6	7	8	9	10	11	12	13	14
1. Extraversion	-													
2. Agreeableness	0.25 ***	-												
3. Conscientiousness	0.22 ***	0.25 ***	-											
4. Emotional Stability	0.18 ***	−0.04	0.23 ***	-										
5. Intellect/Imagination	0.37 ***	0.37 ***	0.31 ***	0.08 *	-									
6. Government malfeasance	0.00	−0.04	−0.02	−0.08 *	−0.02	-								
7. Extraterrestrial cover-up	0.04	−0.14 ***	−0.06	−0.04	−0.09 **	0.47 ***	-							
8. Global conspiracy	0.04	−0.04	0.01	0.08 *	−0.08 *	0.72 ***	0.49 ***	-						
9. Personal wellbeing	0.07 *	−0.00	0.06 *	−0.06	−0.05	0.68 ***	0.53 ***	0.74 ***	-					
10. Information control	0.04	−0.02	0.01	−0.09 **	−0.04	0.69 ***	0.47 ***	0.72 ***	0.74 ***	-				
11. Occurrence beliefs	0.00	0.23 ***	0.04	−0.02	0.20 ***	0.10 **	−0.04	0.00	−0.04	0.06	-			
12. Anthropogenic causation	−0.03	0.31 ***	0.05	−0.08 *	0.19 ***	−0.01	−0.13 * **	−0.07 *	−0.12 ***	−0.01	0.53 ***	-		
13. Gender	−0.00	0.25 ***	0.04	−0.26 ***	0.05	−0.03	−0.09 **	−0.02	−0.04	−0.05	−0.01	0.09 **	-	
14. Age	0.01	−0.00	0.08 **	0.15 ***	−0.01	0.11 **	0.00	0.11 **	0.11 **	0.07 *	−0.00	−0.00	−0.17 ***	-

Notes: * *p* < 0.05; ** *p* < 0.01; *** *p* < 0.001. All gender correlations represent point biserial coefficients.

**Table 3:** Hierarchical linear regression analysis for climate change beliefs dimensions.

Variables	Occurrence Beliefs			Anthropogenic Causation Beliefs		
	B	SE	*β*	B	SE	*β*
*Step 1: Personality traits*						
Extraversion	−0.10	0.03	−0.10 **	−0.10	0.02	−0.14 ***
Agreeableness	0.27	0.04	0.21 ***	0.27	0.03	0.29 ***
Conscientiousness	−0.04	0.04	−0.03	−0.02	0.03	−0.02
Emotional stability	−0.02	0.03	−0.02	−0.03	0.02	−0.04
Imagination/intellect	0.25	0.05	0.18 ***	0.16	0.03	0.15 ***
Age	−0.03	0.15	−0.00	0.02	0.11	0.00
Gender	−1.39	0.57	−0.08 *	0.04	0.42	0.00
*R* ^2^	0.08 ***			0.13 ***		
Δ*R*^2^	0.08 ***			0.13 ***		
*Step 2: Conspiracy beliefs*						
Extraversion	−0.09	0.03	−0.09 *	−0.09	0.02	−0.12 ***
Agreeableness	0.28	0.04	0.22 ***	0.27	0.03	0.29 ***
Conscientiousness	−0.01	0.04	−0.01	−0.00	0.03	−0.00
Emotional stability	−0.02	0.03	−0.02	−0.04	0.02	−0.05
Imagination/intellect	0.21	0.05	0.15 ***	0.13	0.03	0.13 **
Government malfeasance	0.74	0.16	0.24 ***	0.23	0.11	0.10 *
Extraterrestrial cover-up	−0.11	0.12	−0.03	−0.12	0.09	−0.05
Global conspiracy	−0.15	0.17	−0.05	−0.09	0.12	−0.04
Personal wellbeing	−0.75	0.17	−0.24 ***	−0.54	0.12	−0.23 ***
Information control	0.46	0.17	0.14 **	0.39	0.13	0.16 **
Age	−0.06	0.15	−0.01	0.04	0.11	0.01
Gender	−1.39	0.56	−0.08 *	0.00	0.41	0.00
*R* ^2^	0.13 ***			0.16 ***		
Δ*R*^2^	0.04 ***			0.03 ***		

Note. * *p* < 0.05; ** *p* < 0.01; *** *p* < 0.001.

## Data Availability

The data presented in this study are available on request from the corresponding author.

## References

[1] Hickman C., Marks E., Pihkala P., Clayton S., Lewandowski R.E., Mayall E.E., Wray B., Mellor C., van Susteren L. (2021). Climate anxiety in children and young people and their beliefs about government responses to climate change: A global survey. Lancet Planet. Health.

[2] Lee T.M., Markowitz E.M., Howe P.D., Ko C.-Y., Leiserowitz A.A. (2015). Predictors of public climate change awareness and risk perception around the world. Nat. Clim. Chang..

[3] Ebi K.L., Vanos J., Baldwin J.W., Bell J.E., Hondula D.M., Errett N.A., Hayes K., Reid C.E., Saha S., Spector J. (2021). Extreme Weather and Climate Change: Population Health and Health System Implications. Annu. Rev. Public Health.

[4] van der Linden S. (2015). The conspiracy-effect: Exposure to conspiracy theories (about global warming) decreases pro-social behavior and science acceptance. Pers. Individ. Differ..

[5] Leiser D., Wagner-Egger P. (2022). Determinants of Belief–And Unbelief–In Climate Change. Climate of the Middle.

[6] Uscinski J.E., Olivella S. (2017). The conditional effect of conspiracy thinking on attitudes toward climate change. Res. Politics.

[7] Haltinner K., Sarathchandra D. (2021). Predictors of Pro-environmental Beliefs, Behaviors, and Policy Support among Climate Change Skeptics. Soc. Curr..

[8] Uscinski J.E., Douglas K., Lewandowsky S. (2017). Climate change conspiracy theories. Oxford Research Encyclopedia of Climate Science.

[9] Biddlestone M., Azevedo F., van der Linden S. (2022). Climate of conspiracy: A meta-analysis of the consequences of belief in conspiracy theories about climate change. Curr. Opin. Psychol..

[10] Brick C., Lewis G.J. (2014). Unearthing the “Green” Personality. Environ. Behav..

[11] Rothermich K., Johnson E.K., Griffith R.M., Beingolea M.M. (2020). The influence of personality traits on attitudes towards climate change—An exploratory study. Pers. Individ. Differ..

[12] Rahmstorf S. The Climate Sceptics. 2004, Potsdam Institute for Climate Impact Research, Potsdam. http://www.pik-potsdam.de/~stefan/Publications/Other/rahmstorf_climate_sceptics_2004.pdf.

[13] Leiserowitz A., Maibach E., Rosenthal S., Kotcher J., Carman J., Neyens L., Marlon J., Lacroix K., Goldberg M. (2021). Climate Change in the American Mind, 2021, Yale University and George Mason University.

[14] Poortinga W., Bird N., Hallingberg B., Phillips R., Williams D. (2021). The role of perceived public and private green space in subjective health and wellbeing during and after the first peak of the COVID-19 outbreak. Landsc. Urban Plan..

[15] Asplund T. (2014). Natural versus anthropogenic climate change: Swedish farmers’ joint construction of climate perceptions. Public Underst. Sci..

[16] Landon A.C., Woosnam K.M., Keith S.J., Tarrant M.A., Rubin D.M., Ling S.T. (2019). Understanding and modifying beliefs about climate change through educational travel. J. Sustain. Tour..

[17] Mueller J.T., Tickamyer A.R. (2020). Climate change beliefs and support for development: Testing a cognitive hierarchy of support for natural resource-related economic development in rural Pennsylvania. J. Rural. Stud..

[18] Special Eurobarometer 513: Climate Change, 2021. https://europa.eu/eurobarometer/surveys/detail/2273.

[19] Poortinga W., Whitmarsh L., Steg L., Böhm G., Fisher S. (2019). Climate change perceptions and their individual-level determinants: A cross-European analysis. Glob. Environ. Chang..

[20] Balžekiene A., Telešiene A., Telešiene A., Gross M. (2017). Vulnerable and insecure? Environmental and technological risk perception in Europe. Green European.

[21] Patočková D.Č.V. (2020). Individual determinants of climate change scepticism in the Czech Republic. Sociológia.

[22] Nistor L. (2010). The case of the east-central european environmental citizenship in terms of willingness to pay for pollution preven-tion. Stud. Univ. Babes-Bolyai-Sociol..

[23] Nistor L. (2022). The Ranking and Rating of Climate Change in Romania: Trends and Individual-Level Determinants. Sociológia.

[24] McCright A.M., Dunlap R.E., Marquart-Pyatt S.T. (2015). Political ideology and views about climate change in the European Union. Environ. Politics.

[25] Flynn C., Yamasumi E., Fisher S., Snow D., Grant Z., Kirby M., Browning P., Rommerskirchen M., Russell I. (2021). Peoples’ Climate Vote.

[26] Cheval S., Bulai A., Croitoru A.-E., Dorondel Ș., Micu D., Mihăilă D., Sfîcă L., Tișcovschi A. (2022). Climate change perception in Romania. Arch. Meteorol. Geophys. Bioclimatol. Ser. B.

[27] Uscinski J.E., Klofstad C., Atkinson M.D. (2016). What Drives Conspiratorial Beliefs? The Role of Informational Cues and Predispositions. Politics Res. Q..

[28] Enders A.M., Smallpage S.M. (2019). Who Are Conspiracy Theorists? A Comprehensive Approach to Explaining Conspiracy Beliefs. Soc. Sci. Q..

[29] Douglas K.M., Uscinski J.E., Sutton R.M., Cichocka A., Nefes T., Ang C.S., Deravi F. (2019). Understanding Conspiracy Theories. Politics Psychol..

[30] Farmer G.T., Cook J. (2013). Climate Change Science: A Modern Synthesis.

[31] Kerr J.R., Wilson M.S. (2018). Changes in perceived scientific consensus shift beliefs about climate change and GM food safety. PLoS ONE.

[32] Jolley D., Douglas K.M. (2013). The social consequences of conspiracism: Exposure to conspiracy theories decreases intentions to engage in politics and to reduce one’s carbon footprint. Br. J. Psychol..

[33] Lewandowsky S., Gignac G., Oberauer K. (2013). The Role of Conspiracist Ideation and Worldviews in Predicting Rejection of Science. PLoS ONE.

[34] Gifford R., Nilsson A. (2014). Personal and social factors that influence pro-environmental concern and behaviour: A review. Int. J. Psychol..

[35] Kennedy E.H., Krahn H., Krogman N.T. (2013). Are we counting what counts? A closer look at environmental concern, pro-environmental behaviour, and carbon footprint. Local Environ..

[36] Kollmuss A., Agyeman J. (2002). Mind the Gap: Why do people act environmentally and what are the barriers to pro-environmental behavior?. Environ. Educ. Res..

[37] Stern P.C. (2000). Toward a coherent theory of environmentally significant behavior. J. Soc. Issues.

[38] Yu T.-Y., Yu T.-K. (2017). The Moderating Effects of Students’ Personality Traits on Pro-Environmental Behavioral Intentions in Response to Climate Change. Int. J. Environ. Res. Public Health.

[39] Dalvi-Esfahani M., Alaedini Z., Nilashi M., Samad S., Asadi S., Mohammadi M. (2020). Students’ green information technology behavior: Beliefs and personality traits. J. Clean. Prod..

[40] Soutter A.R.B., Bates T.C., Mõttus R. (2020). Big Five and HEXACO Personality Traits, Proenvironmental Attitudes, and Behaviors: A Meta-Analysis. Perspect. Psychol. Sci..

[41] Milfont T.L., Sibley C.G. (2012). The big five personality traits and environmental engagement: Associations at the individual and societal level. J. Environ. Psychol..

[42] Hirsh J.B. (2010). Personality and environmental concern. J. Environ. Psychol..

[43] Jessani Z., Harris P.B. (2018). Personality, politics, and denial: Tolerance of ambiguity, political orientation and disbelief in climate change. Pers. Individ. Differ..

[44] McCrae R.R., John O.P. (1992). An Introduction to the Five-Factor Model and Its Applications. J. Pers..

[45] Gibbon E., Douglas H.E. (2021). Personality and the pro-environmental individual: Unpacking the interplay between attitudes, behaviour and climate change denial. Pers. Individ. Differ..

[46] Markowitz E.M., Goldberg L.R., Ashton M.C., Lee K. (2011). Profiling the “Pro-Environmental Individual”: A Personality Perspective. J. Pers..

[47] Douglas H.E., Bore M., Munro D. (2016). Openness and Intellect: An analysis of the motivational constructs underlying two aspects of personality. Pers. Individ. Differ..

[48] Hirsh J.B., Dolderman D. (2007). Personality predictors of Consumerism and Environmentalism: A preliminary study. Pers. Individ. Differ..

[49] Nisbet E.K., Zelenski J.M., Murphy S.A. (2022). The nature relatedness scale: Linking individuals’ connection with nature to en-vironmental concern and behavior. Environ. Behav..

[50] Kvasova O. (2015). The Big Five personality traits as antecedents of eco-friendly tourist behavior. Pers. Individ. Differ..

[51] Caddick Z.A., Feist G.J. (2021). When beliefs and evidence collide: Psychological and ideological predictors of motivated reasoning about climate change. Think. Reason..

[52] Monday I.F., Sunday I.E. (2020). Climate Change Attitudes, Beliefs and Intentions Among Young Adults in an Institution of Higher Learning: Does Personality Matter?. Int. J. Criminol. Sociol..

[53] Iliescu D., Popa M., Dimache R. (2015). Adaptarea românească a Setului International de Itemide Personalitate: IPIP-Ro [The Ro-manian adaptation of the International Personality Item Pool: IPIP-Ro]. Psihol. Resur. Um..

[54] Pennycook G., Cheyne J.A., Barr N., Koehler D.J., Fugelsang J.A. (2015). On the Reception and Detection of Pseudo-profound Bullshit. Judgm. Decis. Mak..

[55] Brownlee M.T., Verbos R.I. (2015). Measuring outdoor recreationists’ beliefs in climate change: Testing the Occurrence and Anthropogenic Causation Scale (OC-AN). J. Outdoor Recreat. Tour..

[56] Lei M., Lomax R.G. (2005). The effect of varying degrees of nonnormality in structural equation modeling. Struct. Equ. Model..

[57] Coakes S.J. (2005). Analysis without Anguish: Version 12.0 for Windows.

[58] Swami V., Chamorro-Premuzic T., Snelgar R., Furnham A. (2010). Egoistic, altruistic, and biospheric environmental concerns: A path analytic investigation of their determinants. Scand. J. Psychol..

[59] Wood M.J., Douglas K.M., Sutton R.M. (2012). Dead and alive: Beliefs in contradictory conspiracy theories. Soc. Psychol. Personal. Sci..

[60] Kalamas M., Cleveland M., Laroche M. (2013). Pro-environmental behaviors for thee but not for me: Green giants, green Gods, and external environmental locus of control. J. Bus. Res..

[61] Rohrschneider R., Miles M.R. (2015). Representation through parties? Environmental attitudes and party stances in Europe in 2013. Environ. Pol..

[62] Milfont T.L., Milojev P., Greaves L.M., Sibley C.G. (2015). Socio-structural and psychological foundations of climate change beliefs. N. Z. J. Psychol..

[63] Haltinner K., Sarathchandra D., Ptak T. (2021). How Believing That Climate Change Is a Conspiracy Affects Skeptics’ Environmental Attitudes. Environ. Sci. Policy Sustain. Dev..

